# Identity, Belonging, and Psychological Well-Being Among Indigenous Minorities: The Case of Bedouin Young Adults

**DOI:** 10.3390/ijerph23070910

**Published:** 2026-07-16

**Authors:** Nuzha Allassad Alhuzail

**Affiliations:** School of Social Work, Sapir College, Sderot 7956000, Israel; nuzha6@gmail.com

**Keywords:** Bedouin young adults, Indigenous minority, identity, belonging, discrimination, psychological well-being, perceived discrimination

## Abstract

**Highlights:**

**Public health relevance—How does this work relate to a public health issue?**

- This study examines how perceived discrimination, contested belonging, and structural inequality are associated with the psychological well-being of young adults from the Indigenous Bedouin minority in Israel.
- The findings show that well-being is linked not only to individual identity, but also to the social and institutional conditions that enable or undermine recognition, dignity, and belonging.

**Public health significance—Why is this work of significance to public health?**

- The study highlights perceived discrimination and weakened belonging as public-health risk factors that may affect the mental health of young adults in Indigenous and minoritized communities.
- By focusing on Bedouin young adults, the study expands public-health knowledge on the correlations between identity, minority status, structural inequality, and psychological well-being in a context of political and social marginalization.

**Public health implications—What are the key implications or messages for practitioners, policy makers, and/or researchers in public health?**

- Public-health policies and services should move beyond symbolic inclusion and promote culturally responsive, anti-discriminatory, and community-based approaches that strengthen belonging.
- Practitioners, policy makers, and researchers should address discrimination and institutional exclusion as central determinants of mental health, while recognizing Indigenous identity as a potential source of resilience when it is socially affirmed.

**Abstract:**

This cross-sectional study examined identity, belonging, perceived discrimination, and psychological well-being among 348 Bedouin young adults in Israel (ages 18–30; M = 20.8, SD = 3.7). The survey assessed identity endorsement and centrality, belonging to the Bedouin community, perceived discrimination, perceived identity threats, and psychological well-being. Latent class analysis identified three descriptive identity profiles: (1) Ambivalent/Moderate Bedouin (mixed identities with moderate endorsement; *n* = 158), (2) Bedouin-Centered (near-exclusive endorsement of Bedouin identity with limited endorsement of the other measured categories; *n* = 74), and (3) Integrated Religious-National Bedouin (high Bedouin, Arab, and Muslim endorsement; *n* = 116). The Integrated profile reported somewhat lower well-being, higher perceived discrimination, and more perceived identity threats in descriptive comparisons. In hierarchical regression, stronger belonging was associated with higher well-being and greater perceived discrimination with lower well-being after adjustment for demographics; identity profiles did not significantly improve prediction once these social experiences were included. The findings underscore that psychological well-being among Indigenous minority young adults is associated not only with identity configuration but also with the social conditions that enable belonging and reduce discrimination.

## 1. Introduction: Identity, Belonging, and Well-Being in the Bedouin Context

Identity formation continues throughout young adulthood and is shaped by social position, institutional encounters, and the political meanings attached to group membership. For Indigenous minority young adults, identity often involves negotiating heritage culture, community attachment, national citizenship, religion, and relations with the dominant society. Social identity theory suggests that socially recognized group identities can support self-concept and well-being, whereas stigmatization, identity threat, and discrimination may contribute to psychological distress [1].

Research with young Indigenous people similarly emphasizes the importance of cultural continuity, community connection, and social recognition for mental well-being, while historical and contemporary inequalities may undermine these resources [2,3]. In this study, psychological well-being refers to participants’ self-reported positive affect, life satisfaction, self-rated mental health, and lower emotional distress. It is therefore a psychological outcome rather than a clinical diagnosis or a measure of physical health; the broader term “health” is used only in the public-health sense.

The Negev Bedouin are an Indigenous Arab minority in southern Israel whose social world has been reshaped by sedentarization, rapid socioeconomic change, and continuing inequalities in access to land, services, education, and employment [4,5]. These conditions make identity and belonging especially salient for young adults who navigate community expectations alongside engagement with state institutions and wider society.

Bedouin young adults may identify simultaneously with local Bedouin and tribal affiliations, Arab and Palestinian national identities, Israeli civic identity, and Muslim religious identity. The salience and compatibility of these affiliations may vary across social settings and may be shaped by education, gender, urbanization, and experiences of exclusion [6,7,8].

This study examines correlations between identity configurations, belonging, perceived discrimination, and psychological well-being among Bedouin young adults. It has three objectives: (1) to identify common identity profiles through latent class analysis; (2) to test whether belonging and perceived discrimination are associated with psychological well-being after adjustment for demographic characteristics; and (3) to describe whether the profiles differ in the extent of belonging, discrimination, perceived identity threats, and well-being. We expected stronger belonging to be associated with higher psychological well-being and greater perceived discrimination to be associated with lower psychological well-being. Because quantitative evidence on identity-profile differences in this population is limited, comparisons among profiles are treated as exploratory.

## 2. Materials and Methods

### 2.1. Participants and Procedure

This cross-sectional study included 348 Bedouin Arab young adults from the Negev region of Israel. Eligibility criteria required Bedouin descent and age 18–30 years; all participants were adults. Detailed demographic characteristics are presented in the Results section to distinguish the study design and recruitment procedures from empirical findings.

Participants were recruited through community centers and local organizations in Bedouin localities, including community-based educational, youth, and civil society frameworks. After informed consent was obtained, participants completed an Arabic questionnaire administered in person by trained research assistants from the Bedouin community. Respondents were promised anonymity in any publication of the research and that their responses would be used only for research purposes. Ethical approval was obtained from the researchers’ academic institution.

### 2.2. Measures

All key constructs were assessed through self-report items. The questionnaire combined identity items with measures that were adapted from existing minority and well-being research and, where no directly suitable standardized instrument was available, items developed for the Bedouin context with expert consultation. It was administered in Arabic. The section below specifies the construct, response format, scoring, and illustrative wording for each measure. Internal consistency is reported for multi-item scales. Because formal psychometric validation of the adapted or newly developed measures was not conducted in this study, they should be understood as context-specific exploratory measures rather than diagnostic instruments.

#### 2.2.1. Identity Endorsement

To capture multiple facets of identity, participants were presented with six identity categories—Bedouin, Arab, Palestinian, Israeli, Muslim, and tribal/clan—and were asked to select all identities that applied to them. Each selected identity was coded 1, and each unselected identity 0. A count of endorsed identities (0–6) was also calculated to describe identity breadth. This multiple-response format was used because Bedouin young adults may hold several group identities simultaneously.

#### 2.2.2. Identity Centrality

For each of the same six identity domains, participants rated centrality to their daily life on a five-point Likert scale (1 = not at all central to who I am; 5 = extremely central to who I am). The item stem was “My [identity] identity is central to my sense of self”, adapted for Bedouin, Arab, Palestinian, Israeli, Muslim, and tribal identities. These are six distinct single-item indicators rather than a combined scale. They were used descriptively to contextualize identity salience and were not entered into the latent class or regression models because of their conceptual overlap with identity endorsement.

#### 2.2.3. Sense of Belonging

Sense of belonging to the Bedouin community was assessed using a three-item, context-specific scale inspired by existing measures of belongingness. The items addressed perceived membership, togetherness, and connectedness to the Bedouin community; illustrative items were “I feel that I belong to the Bedouin community” and “I have a strong sense of togetherness with other Bedouin people.” Responses ranged from 1 (strongly disagree) to 5 (strongly agree). Item scores were averaged (Cronbach’s α = 0.71), with higher scores indicating stronger perceived belonging.

#### 2.2.4. Perceived Discrimination

Perceived discrimination was assessed with four adapted items that asked how often participants felt they were treated unfairly or negatively because they are Bedouin. The items focused on perceived discriminatory treatment in everyday life; an illustrative item was “I feel that people have discriminated against me because I am Bedouin.” Responses ranged from 1 (never) to 5 (very often). Item scores were averaged (Cronbach’s α = 0.69), with higher scores reflecting more frequent perceived discrimination.

#### 2.2.5. Psychological Well-Being

Psychological well-being was operationalized as a composite of 17 self-report indicators covering positive and negative affect, life satisfaction, and self-rated general mental health. Two evaluative items were rated from 0 to 10, and 15 affective items were rated from 1 to 6. Each item was linearly transformed to a 0–1 range before averaging, so that the different response scales contributed equally. The resulting index (Cronbach’s α = 0.81) ranges from 0 to 1, with higher scores indicating more positive psychological well-being and lower emotional distress. It is not a clinical diagnostic measure and does not represent physical health.

#### 2.2.6. Perceived Identity Threats

Items were included to assess whether participants see various forces as threats to the Bedouin identity or way of life. They were presented with a list of seven potential “identity threats” derived from community input, and literature on challenges faced by Bedouins: (1) government policies (such as land confiscation, relocation plans, home demolitions); (2) institutional treatment (discrimination or neglect by state institutions such as schools, police, healthcare); (3) social media and outside cultural influence (erosion of traditions via modern media); (4) poverty and lack of equality (socio-economic marginalization); (5) pressure to merge or assimilate into mainstream society (losing distinct identity); (6) conflicts within Bedouin families/communities (internal disputes, generational tensions weakening identity); and (7) harm to the Arabic language (diminishing use of Arabic/Bedouin dialect). Participants checked which of these they perceived as real threats to the continuity of Bedouin identity. This enabled calculation of the number of threats perceived (0 to 7) for each person, and the percentage of participants endorsing each threat was also noted. Additionally, a summary statement on a 5-point scale asked whether “Overall, threats to Bedouin culture make it hard for me to express my identity,” to capture the subjective impact of these threats (with 5 indicating that participants strongly agreed that it weakens their identity expression).

### 2.3. Data Analysis

The analysis proceeded in several steps:

#### 2.3.1. Descriptive Statistics

Descriptive statistics were examined for all variables, including means, standard deviations, and proportions. This produced an overview of the sample’s characteristics (e.g., average well-being, average belonging, distribution of identity endorsements). Internal reliability (Cronbach’s α) was checked for the multi-item scales (belonging, discrimination, well-being).

#### 2.3.2. Latent Class Analysis (LCA) of Identity Profiles

Latent class analysis (LCA) was conducted using the six dichotomous identity-endorsement variables (Bedouin, Arab, Palestinian, Israeli, Muslim, and tribal/clan) in the poLCA package in R (version 4.5.2). Models with one through six classes were examined. Selection of the three-class solution was based on the combined consideration of model-fit indices (AIC, BIC, adjusted BIC, and entropy), class sizes, theoretical interpretability, and the usefulness of the resulting profiles for describing the data. Participants were assigned to the class with the highest posterior membership probability. Because the study is cross-sectional and the profiles are data-driven, the LCA is interpreted as exploratory and descriptive rather than as a definitive typology.

#### 2.3.3. Differences Across Identity Profiles

Profile differences in psychological well-being, perceived discrimination, sense of belonging, perceived identity threats, and demographic characteristics were examined using the tests described below. Kruskal–Wallis tests were used for continuous or ordinal outcomes because some distributions departed from normality, and several measures were based on Likert-type response formats. Significant omnibus tests were followed, where relevant, by Bonferroni-adjusted pairwise comparisons. Pearson’s chi-square tests, or Fisher’s exact tests when expected cell counts were small, were used for categorical variables. The exploratory regression models for sense of belonging and perceived discrimination are presented in Table 1 and Table 2, respectively.

#### 2.3.4. Correlation Analysis

As a preliminary check of correlations between variables, Pearson correlation coefficients were computed between the main continuous variables: well-being, belonging, discrimination, number of identities, and number of threats. This helped identify basic associations (e.g., whether belonging correlates with well-being) and multicollinearity issues for regression.

#### 2.3.5. Hierarchical Multiple Regression (HMR) for Well-Being

To examine predictors of psychological well-being and to test the incremental value of identity profiles, a hierarchical regression was performed according to three models:

Model 1: Included only demographic variables as predictors (age, gender, residence type dummies, education, marital status, living with parents). This established a baseline and controls for basic factors.

Model 2: Added two key psychosocial variables—perceived discrimination and sense of belonging—and also the number of identity threats. These were added to understand the extent to which subjective experiences explained variance in well-being beyond demographics.

Model 3: Added two dummy variables representing the identity profile (with the largest class as reference). This tested whether knowing a person’s identity profile category improved the prediction of well-being, after accounting for discrimination, belonging, and demographics.

R^2^ changes, F-change tests, and regression coefficients were examined at each step. The aim was to examine whether identity profiles explained additional variance in well-being beyond demographic variables, belonging, discrimination, and perceived identity threats. Because the design was cross-sectional, the regression models were not interpreted as tests of mediation or causality.

#### 2.3.6. Ancillary Regression Analyses (Belonging and Discrimination)

Ancillary regression analyses were conducted for belonging and perceived discrimination to describe exploratory associations with demographic characteristics and profile membership. Candidate predictors were first examined in univariable models; multivariable models included predictors associated at *p* < 0.05. AIC-based subset selection was then performed, but only to present a parsimonious exploratory model. Because stepwise procedures can be unstable and data-driven, these results are interpreted cautiously and are not treated as confirmatory evidence.

All analyses were conducted in R (Version 4.5.2). Statistical tests were two-tailed with α = 0.05. Results are presented with effect estimates and 95% confidence intervals where applicable. Given the cross-sectional design, all reported correlations are interpreted as associations rather than causal effects.

## 3. Results

### 3.1. Sample Characteristics

The final sample comprised 348 Bedouin young adults aged 18–30 years (M = 20.8, SD = 3.7). Of the participants, 137 (39%) were male and 211 (61%) were female. Most were single (76%) and lived with their parents (93%). Fifty-four percent had pursued or completed academic higher education, whereas the remainder had high school or vocational education. Approximately 85% of mothers and 86% of fathers had no academic education. Participants lived in government-planned urban Bedouin towns (28%), recognized rural villages or tribal communities (47%), and unrecognized villages (25%). Because recruitment relied on community and organizational networks, and the sample included a relatively high proportion of academically educated participants, it should not be interpreted as representative of all Bedouin young adults.

### 3.2. Overall Identity, Belonging, Discrimination, and Well-Being Patterns

Participants endorsed an average of 2.28 identity categories (SD = 1.36; range = 1–6), indicating that multiple group identities were common. Bedouin identity was endorsed by 64% of participants, Muslim identity by 63%, Arab identity by 51%, Palestinian identity by 33%, and Israeli identity by 6%. These descriptive figures show that identity endorsement in the sample was diverse and often overlapping.

Participants reported a mean sense of belonging to Bedouin society of 3.61 (SD = 1.07) on a 1–5 scale and a mean perceived-discrimination score of 2.80 (SD = 0.98). Mean psychological well-being was 0.51 (SD = 0.17) on the 0–1 composite index. Participants perceived an average of 2.25 identity threats (SD = 0.90); government policy was the most frequently selected threat (52.9%), followed by social media and external influences (43.4%) and institutional treatment (36.8%). These results describe the present sample and should not be interpreted as population-wide estimates.

### 3.3. Identity Profiles from Latent Class Analysis

**Figure 1:**
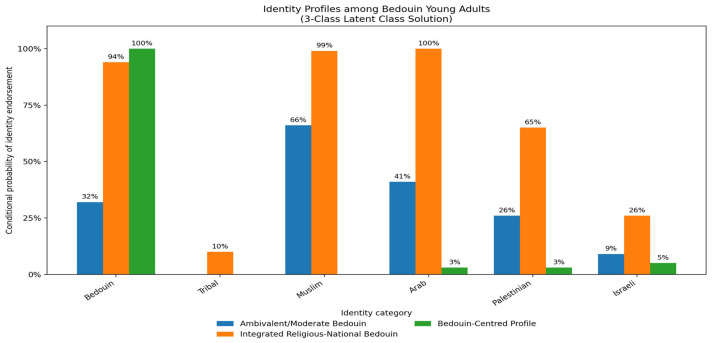
Conditional probabilities of identity endorsement across the three latent profiles.

Latent class analysis identified three identity-endorsement profiles. Figure 1 presents the conditional probability of endorsing each identity category within the three profiles. The profile labels are descriptive summaries of response patterns rather than fixed social categories.

#### 3.3.1. Profile 1: Ambivalent/Moderate Bedouin

This was the largest group, comprising 45.4% of the sample (*n* = 158). Young adults in this profile had a mixed identity pattern with relatively lower endorsement across identities compared to other profiles. According to the LCA, individuals in this class were characterized by about one-third endorsing Bedouin identity (32% probability), and about two-thirds endorsing Muslim identity (66%). For broader identities, around 41% identified as Arab and 26% as Palestinian, and 9% as Israeli. This profile was labeled Ambivalent/Moderate Bedouin because it reflects moderate or uneven endorsement across categories rather than strong commitment to a single identity pattern.

#### 3.3.2. Profile 2: Bedouin-Centered Profile

This profile represented 21.3% of participants (*n* = 74). It was characterized by near-universal endorsement of Bedouin identity and limited endorsement of the other measured identity categories. We use the term Bedouin-Centered Profile rather than “non-religious” to avoid treating a data-derived response pattern as a fixed social or religious identity. The profile should therefore be interpreted cautiously as a statistical configuration of endorsements within this sample.

#### 3.3.3. Profile 3: Integrated Religious-National Bedouin

This profile represented 33.3% of the sample (*n* = 116). These individuals demonstrated a broad, multifaceted identity, endorsing multiple categories at high rates. Most notably, this class had a near 100% probability of endorsing Arab identity (100%), Muslim identity (99%), and Bedouin identity (94%). A majority also identified as Palestinian (about 65%), and around a quarter as Israeli (26%). This profile was labeled Integrated Religious-National Bedouin because it combined Bedouin ethnic identity with Muslim religious identity and broader Arab-Palestinian national identification.

### 3.4. Differences Across Identity Profiles

The following analyses compare the three profiles on psychological well-being, perceived discrimination, belonging, perceived identity threats, and demographic characteristics. These comparisons are exploratory and reflect associations within this sample.

#### 3.4.1. Psychological Well-Being

Psychological well-being differed across profiles (Kruskal–Wallis *p* = 0.008). The Integrated Religious-National profile reported the lowest mean well-being (approximately 0.48 on the 0–1 scale), whereas the Ambivalent/Moderate and Bedouin-Centered profiles both reported means of approximately 0.53. Bonferroni-adjusted post hoc comparisons indicated lower well-being in the Integrated profile than in the other two profiles. Because mean differences were modest and the design was cross-sectional, this pattern should be interpreted cautiously.

#### 3.4.2. Perceived Discrimination

Perceived discrimination differed across profiles (*p* = 0.046). The Integrated Religious-National profile reported the highest mean discrimination (approximately 2.96 on the 1–5 scale), the Ambivalent/Moderate profile the lowest (approximately 2.67), and the Bedouin-Centered profile an intermediate mean (approximately 2.80). The Integrated profile reported more frequent perceived discrimination than the Ambivalent/Moderate profile. The analysis does not establish why these differences occurred.

#### 3.4.3. Sense of Belonging

Differences in belonging across profiles were marginally significant (*p* = 0.050). The Bedouin-Centered profile reported the highest mean community belonging (approximately 3.81 on the 1–5 scale), compared with approximately 3.57 for the Integrated Religious-National profile and 3.55 for the Ambivalent/Moderate profile. Given the marginal *p*-value and the exploratory nature of the comparison, this finding is interpreted cautiously.

#### 3.4.4. Perceived Identity Threats

The profiles differed in the number of perceived identity threats (*p* = 0.013), with the Integrated Religious-National profile reporting the highest mean number (approximately 2.46) compared with approximately 2.14 in the other profiles. At the item level, the Integrated profile was more likely to identify government policy as a threat (66% versus 43% and 54% for the other profiles; *p* = 0.001) and showed a trend toward greater endorsement of institutional treatment as a threat (46% versus 32% and 32%; *p* = 0.051).

The distribution of specific perceived identity threats across the three identity profiles is presented in Table 3. The hierarchical regression results for psychological well-being are presented in Table 4.

### 3.5. Demographics (Gender and Education)

Gender and education also differed across profiles. The Integrated Religious-National profile included a higher proportion of female and academically educated participants, whereas the Bedouin-Centered profile included a higher proportion of male participants and a lower proportion of academically educated participants. Age did not differ significantly across profiles.

In summary, the profiles were associated with differences in reported experiences and demographic composition. The Integrated Religious-National profile reported somewhat lower well-being, higher perceived discrimination, and more perceived identity threats, while the Bedouin-Centered profile reported the highest belonging. These findings are descriptive associations and do not imply that identity configurations cause differences in well-being.

## 4. Discussion

This study examined associations between identity configurations, belonging, perceived discrimination, and psychological well-being among Bedouin young adults. Three findings provide the main takeaway. First, stronger community belonging was associated with higher psychological well-being, whereas perceived discrimination was associated with lower well-being after adjustment for demographic characteristics. Second, the Integrated Religious-National profile reported somewhat lower well-being and more discrimination and identity threats in descriptive comparisons. Third, identity profiles did not add significant predictive value once belonging, discrimination, and demographics were considered. Taken together, the findings suggest that social recognition, inclusion, and everyday experiences of discrimination are more proximal correlates of psychological well-being than identity labels alone.

### 4.1. Belonging and Discrimination as Factors Associated with Well-Being

A key contribution of this study is the finding that sense of belonging and perceived discrimination were more strongly associated with psychological well-being than identity profiles themselves. This aligns with extensive evidence highlighting belonging as a fundamental human need and a critical determinant of mental health across cultures [9,10]. Recent scholarship further emphasizes that for Indigenous and minoritized young people, belonging is deeply relational and collective, rooted in connection to community, culture, and shared history [2,11,12].

At the same time, perceived discrimination was independently associated with diminished well-being. Discrimination functions as a chronic psychosocial stressor that communicates social devaluation and exclusion, thereby undermining self-worth and psychological safety [13,14]. Contemporary minority stress frameworks emphasize that repeated exposure to discrimination accumulates over time, contributing to emotional exhaustion, anxiety, and depressive symptoms [15]. For Bedouin young adults, discrimination is embedded not only in interpersonal encounters but also in structural conditions, including unequal access to land, services, and educational opportunities; factors increasingly recognized as central to Indigenous mental health disparities [16,17].

Importantly, the independent effects of belonging and discrimination indicate that they are not simply opposite poles of a single continuum. Strong community belonging does not fully buffer against the psychological costs of discrimination, nor does low exposure to discrimination compensate for weak belonging. This finding reinforces recent calls to conceptualize well-being among Indigenous youth as shaped by multiple, intersecting social processes rather than by identity alone [18,19].

### 4.2. Identity Profiles and the Importance of Sociopolitical Context

The analysis of identity profiles indicates that multiple identity affiliations are not uniformly protective in settings marked by political conflict and unequal power relations. While acculturation theory often frames integration as adaptive [20], the current findings suggest that the psychological implications of integrated identities depend on whether these identities are socially recognized and can be expressed without discrimination. These descriptive patterns should not be interpreted as evidence that integrated identities are harmful.

For Bedouin young adults, the Integrated Religious-National profile was associated with higher perceived discrimination and identity-related threats. One possible interpretation is that participants who are more visibly engaged across community, religious, national, and institutional domains may encounter greater awareness of exclusion or inequality. This is an interpretation rather than a causal explanation: the cross-sectional design cannot determine whether identity configuration precedes discrimination, whether discrimination shapes identity salience, or whether both reflect unmeasured social experiences.

The Bedouin-Centered profile reported relatively high community belonging and well-being, similar to the Ambivalent/Moderate profile. This profile should not be interpreted as evidence of a stable “non-religious” subgroup or as a preferred identity pathway. It is a data-derived pattern characterized by strong Bedouin endorsement and limited endorsement of the other categories measured here. Its emergence requires replication with larger and more diverse samples, qualitative inquiry, and measures that capture the meanings participants attach to their identities.

The Ambivalent/Moderate profile further illustrates the contextual nature of identity processes. Participants in this profile had lower or uneven endorsement across the measured identities, yet they did not report the lowest well-being. This pattern may reflect developmental exploration, situational flexibility, or measurement limits rather than a coherent identity status. Longitudinal and qualitative research is required to examine these possibilities.

Importantly, identity profiles did not significantly improve the prediction of psychological well-being, once belonging and discrimination were included. This result is consistent with the view that identity is linked to well-being, in part, through the social positions, opportunities, and experiences that accompany it. In this study, identity appeared most consequential when considered together with whether participants felt accepted by their community and whether they encountered discriminatory treatment.

### 4.3. Education, Mobility, and the Integration Paradox

The findings also illustrate the integration paradox whereby higher educational attainment is associated with increased perceptions of discrimination and reduced belonging. This phenomenon has been documented among immigrant and minority populations in Europe and North America [21,22] and is increasingly recognized among Indigenous youth navigating mainstream institutions [17].

For Bedouin young adults, academic mobility often entails entry into Jewish-majority educational spaces, where cultural difference becomes more salient and belonging more fragile. Simultaneously, time spent away from home communities may weaken traditional sources of social support. While education remains a crucial pathway to long-term socioeconomic well-being, these findings highlight the possible emotional and psychological costs of upward mobility in unequal societies [23]. Transitional supports, culturally responsive campuses, and institutional accountability for discrimination are therefore essential components of equity-oriented educational policy.

### 4.4. Gender, Intersectionality, and Differential Belonging

Although gender did not emerge as a strong direct predictor of well-being, gendered patterns across identity, belonging, and perceived threats were evident. Young Bedouin women were more likely to occupy integrated identity positions and to report lower belonging, suggesting a compounded vulnerability shaped by both gender and ethnic minority status. Intersectionality theory provides a useful lens for understanding how overlapping systems of patriarchy and ethnonational marginalization structure these experiences [24,25].

Research on Indigenous and minority women highlights that educational and social mobility often entails negotiating tensions between empowerment and social sanction [26,27]. The findings underscore the need for gender-sensitive approaches to strengthening belonging, including creating spaces that recognize women’s evolving roles and contributions within their communities.

## 5. Limitations and Future Directions

Several limitations should be acknowledged. First, the cross-sectional design does not permit causal conclusions about the correlations between identity, belonging, discrimination, and psychological well-being. Second, the non-probability sample was not representative of the wider Bedouin population and included a relatively high proportion of participants connected to academic or community-based frameworks. Third, the study did not collect systematic information on potentially relevant experiences, such as military service, social class, local context, or exposure to particular state institutions. Fourth, several measures were adapted or developed for this study. Although their internal consistencies were acceptable, formal psychometric validation, including factor-structure and measurement-invariance testing, was not conducted. Finally, the data-driven identity profiles and AIC-based ancillary models require replication before they should be generalized.

### Future Directions

Future research should use longitudinal and mixed-method designs to examine how identity meanings, discrimination, belonging, and psychological well-being change over time. It should validate culturally grounded Arabic measures in partnership with Bedouin communities, report complete latent-class model-comparison statistics, and test whether the profiles are replicated in larger, more diverse samples. Qualitative research would be especially valuable for understanding how participants themselves interpret the identity configurations identified here and to identify protective practices in families, communities, educational institutions, and health services.

## 6. Conclusions

The findings support a public-health approach that treats belonging and discrimination as social determinants of psychological well-being among Bedouin young adults. Practical implications include community-led and culturally responsive mental-health initiatives, institutional anti-discrimination practices, equitable access to education and services, and settings in which young adults can express Indigenous identity without social penalty. Rather than prescribing a single identity pathway, policy and practice should strengthen the conditions under which diverse identities can be recognized, supported, and connected to meaningful community belonging.

## Figures and Tables

**Table 1:** Exploratory regression models for sense of belonging.

	Univariable Model			Multivariable Model			Best AIC		
Characteristic	Beta	95% CI	*p*-Value	Beta	95% CI	*p*-Value	Beta	95% CI	*p*-Value
**Age**	0.00	−0.03, 0.03	0.950	−0.03	−0.07, 0.02	0.251			
**Gender**			0.064			0.049			0.092
Male	—	—		—	—		—	—	
Female	−0.22	−0.45, 0.01		−0.28	−0.55, 0.00		−0.20	−0.43, 0.03	
**Education**			0.032			0.230			0.045
Non-academic	—	—		—	—		—	—	
Academic	−0.25	−0.47, −0.02		−0.17	−0.44, 0.11		−0.23	−0.46, −0.01	
**Marital status**			0.153			0.030			
Single	—	—		—	—				
Other	0.19	−0.07, 0.46		0.43	0.04, 0.81				
**Residence type**			0.170			0.224			
Planned Urban Towns	—	—		—	—				
Recognized Rural/Tribal/other	0.24	−0.03, 0.51		0.27	−0.04, 0.57				
Unrecognized village	0.24	−0.07, 0.55		0.17	−0.19, 0.52				
**Living with parents**			0.908			0.825			
Yes	—	—		—	—				
No	−0.03	−0.46, 0.41		0.07	−0.51, 0.65				
**Paternal education**			0.047			0.065			
Non-academic	—	—		—	—				
Academic	−0.34	−0.68, 0.00		−0.37	−0.76, 0.02				
**Maternal education**			0.630			0.982			
Non-academic	—	—		—	—				
Academic	−0.09	−0.44, 0.27		0.00	−0.38, 0.37				

**Table 2:** Exploratory regression models for perceived discrimination.

	Univariable Model			Multivariable Model			Best AIC		
Characteristic	Beta	95% CI	*p*-Value	Beta	95% CI	*p*-Value	Beta	95% CI	*p*-Value
**Age**	0.04	0.02, 0.07	0.002	0.03	0.00, 0.07	0.084	0.04	0.01, 0.06	0.011
**Gender**			0.565			0.788			
Male	—	—		—	—				
Female	0.06	−0.15, 0.27		0.03	−0.21, 0.28				
**Education**			0.004			0.193			0.013
Non-academic	—	—		—	—		—	—	
Academic	0.30	0.10, 0.51		0.16	−0.08, 0.40		0.26	0.05, 0.46	
**Marital status**			0.242			0.985			
Single	—	—		—	—				
Other	0.14	−0.10, 0.39		0.00	−0.35, 0.34				
**Residence type**			0.030			0.060			0.096
Planned Urban Towns	—	—		—	—		—	—	
Recognized Rural/Tribal/other	−0.32	−0.56, −0.07		−0.33	−0.60, −0.06		−0.26	−0.50, −0.02	
Unrecognized village	−0.29	−0.58, −0.01		−0.22	−0.54, 0.10		−0.23	−0.51, 0.05	
**Living with parents**			0.805			0.603			
Yes	—	—		—	—				
No	−0.05	−0.45, 0.35		−0.14	−0.66, 0.38				
**Paternal education**			0.489			0.982			
Non-academic	—	—		—	—				
Academic	0.11	−0.20, 0.42		0.00	−0.36, 0.35				
**Maternal education**			0.354			0.293			
Non-academic	—	—		—	—				
Academic	0.15	−0.17, 0.47		0.18	−0.16, 0.51				

**Table 3:** Identity threats by profile.

Characteristic	Overall *N* = 348 ^1^	Ambivalent/Moderate Bedouin *n* = 158 ^1^	Bedouin-Centered Profile *n* = 74 ^1^	Integrated Religious-National Bedouin *n* = 116 ^1^	*p*-Value ^2^
Government policy	184 (53%)	68 (43%)	40 (54%)	76 (66%)	0.001
Institutional treatment	128 (37%)	51 (32%)	24 (32%)	53 (46%)	0.051
Social media and external influences	151 (43%)	66 (42%)	29 (39%)	56 (48%)	0.401
Poverty and lack of equal opportunities	77 (22%)	39 (25%)	14 (19%)	24 (21%)	0.554
Pressure to merge/assimilate	73 (21%)	37 (23%)	12 (16%)	24 (21%)	0.453
Conflicts within family/community	74 (21%)	33 (21%)	18 (24%)	23 (20%)	0.752
Harm to the Arabic language	95 (27%)	44 (28%)	22 (30%)	29 (25%)	0.758
**Threats weaken the ability to express identity, Mean (SD)**	2.94 (1.35)	2.86 (1.35)	2.84 (1.58)	3.11 (1.17)	0.242

^1^ *n* (%). ^2^ Pearson’s chi-squared test or Kruskal–Wallis rank-sum test, as appropriate.

**Table 4:** Hierarchical linear regression for well-being.

	Model 1: Demographics			Model 2: Discrimination, Belonging, and Identity Threats			Model 3: Identity Profiles		
Characteristic	Beta	95% CI	*p*-Value	Beta	95% CI	*p*-Value	Beta	95% CI	*p*-Value
**Age**	0.00	−0.01, 0.00	0.767	0.00	0.00, 0.01	0.549	0.00	0.00, 0.01	0.544
**Residence type**			0.964			0.685			0.640
Planned Urban Towns	—	—		—	—		—	—	
Recognized Rural/Tribal/other	0.01	−0.04, 0.05		−0.02	−0.06, 0.02		−0.02	−0.06, 0.02	
Unrecognized village	0.00	−0.05, 0.05		−0.02	−0.06, 0.03		−0.02	−0.06, 0.03	
**Gender**			0.030			0.104			0.132
Male	—	—		—	—		—	—	
Female	−0.04	−0.08, 0.00		−0.03	−0.06, 0.01		−0.03	−0.06, 0.01	
**Education**			0.175			0.843			0.968
Non-academic	—	—		—	—		—	—	
Academic	−0.03	−0.06, 0.01		0.00	−0.04, 0.03		0.00	−0.03, 0.03	
**Marital status**			0.215			0.049			0.044
Single	—	—		—	—		—	—	
Other	−0.03	−0.09, 0.02		−0.05	−0.10, 0.00		−0.05	−0.10, 0.00	
**Living with parents**			0.055			0.035			0.028
Yes	—	—		—	—		—	—	
No	0.08	0.00, 0.15		0.07	0.01, 0.14		0.08	0.01, 0.15	
**Belonging**				0.05	0.03, 0.07	<0.001	0.05	0.03, 0.07	<0.001
**Discrimination**				−0.04	−0.06, −0.02	<0.001	−0.04	−0.06, −0.02	<0.001
**Number of threats**				−0.01	−0.03, 0.01	0.269	−0.01	−0.03, 0.01	0.399
**Identity profile**									0.134
Ambivalent/Moderate Bedouin							—	—	
Bedouin-Centered Profile							−0.01	−0.05, 0.03	
Integrated Religious-National Bedouin							−0.04	−0.08, 0.00	

## Data Availability

The data are not publicly available due to confidentiality and ethical restrictions concerning a small Indigenous minority community. De-identified data may be made available from the corresponding author upon reasonable request and subject to ethical approval.

## References

[1] Yip T., Gee G.C., Takeuchi D.T. (2009). Racial discrimination and psychological distress: The impact of ethnic identity and age among immigrant and United States-born Asian adults. Dev. Psychol..

[2] Snowshoe A., Crooks C.V., Tremblay P.F., Hinson R.E. (2017). Cultural connectedness and its relation to mental wellness for First Nations youth. J. Prim. Prev..

[3] Anderson K., Elder-Robinson E., Gall A., Ngampromwongse K., Connolly M., Letendre A., Willing E., Akuhata-Huntington Z., Howard K., Dickson M. (2022). Aspects of wellbeing for Indigenous Youth in CANZUS countries: A systematic review. Int. J. Environ. Res. Public Health.

[4] Allassad Alhuzail N., Besser A., Zeigler-Hill V. (2024). Sharing your husband: Adult attachment styles and emotional responses of Israeli Bedouin-Arab women to potential polygynous marriage. Int. J. Environ. Res. Public Health.

[5] Allassad Alhuzail N., Mahajne I. (2025). Balancing acts: The intricate role of child protection officers in empowering Bedouin girls for gender equality in patriarchal families. J. Ethn. Cult. Divers. Soc. Work.

[6] Abu-Saad I., Yonah Y., Kaplan A. (2000). Identity and political stability in an ethnically diverse state: A study of Bedouin Arab youth in Israel. Soc. Identities.

[7] Parizot C. (2001). Gaza, Beersheba, Dhahriyya: Another approach to the Negev Bedouins in the Israeli-Palestinian Space. Bull. Cent. Rech. Fr. Jérusalem.

[8] Allassad Alhuzail N. (2023). I just live in the village, but I don’t belong to it: Educated young Bedouin men and belonging. J. Hous. Built Environ..

[9] Baumeister R.F., Leary M.R. (1995). The need to belong: Desire for interpersonal attachments as a fundamental human motivation. Psychol. Bull..

[10] Lambert N.M., Stillman T.F., Hicks J.A., Kamble S., Baumeister R.F., Fincham F.D. (2013). To belong is to matter: Sense of belonging enhances meaning in life. Pers. Soc. Psychol. Bull..

[11] Kirmayer L.J., Gone J.P., Moses J. (2014). Rethinking historical trauma. Transcult. Psychiatry.

[12] Allassad Alhuzail N. (2021). The social representation of the Bedouin woman. Women’s Stud. Int. Forum.

[13] Pascoe E.A., Smart Richman L. (2009). Perceived discrimination and health: A meta-analytic review. Psychol. Bull..

[14] Williams A.D., Clark T.C., Lewycka S. (2019). The associations between cultural identity and mental health outcomes for indigenous Māori youth in New Zealand. Front. Public Health.

[15] Meyer I.H. (2015). Resilience in the study of minority stress and health of sexual and gender minorities. Psychol. Sex. Orientat. Gend. Divers..

[16] Reading C.L., Wien F. (2009). Health Inequalities and Social Determinants of Aboriginal Peoples’ Health.

[17] Hatala A.R., Morton D., Njeze C., Bird-Naytowhow K., Pearl T. (2020). Re-imagining miyo-wicehtowin: Human-nature relations, land-making, and wellness among Indigenous youth in a Canadian urban context. Soc. Sci. Med..

[18] Allen J., Hopper K., Wexler L., Kral M., Rasmus S., Nystad K. (2014). Mapping resilience pathways of Indigenous youth in five circumpolar communities. Transcult. Psychiatry.

[19] Burnette C.E., Figley C.R. (2017). Historical oppression, resilience, and transcendence: Can a holistic framework help explain violence experienced by Indigenous people?. Soc. Work.

[20] Berry J.W. (1997). Immigration, acculturation, and adaptation. Appl. Psychol..

[21] Verkuyten M. (2016). The integration paradox: Empiric evidence from the Netherlands. Am. Behav. Sci..

[22] Steinmann J.-P. (2019). The paradox of integration: Why do higher educated new immigrants perceive more discrimination in Germany?. J. Ethn. Migr. Stud..

[23] Ferguson I., Ioakimidis V., Lavalette M. (2018). Global Social Work in a Political Context. Radical Perspectives.

[24] Crenshaw K. (1991). Mapping the margins: Intersectionality, identity politics, and violence against women of color. Stanf. Law Rev..

[25] Else-Quest N.M., Hyde J.S. (2016). Intersectionality in quantitative psychological research: I. Theoretical and epistemological issues. Psychol. Women Q..

[26] Allassad Alhuzail N., Zanoon A., Alhujjaj M. (2026). Indigenous knowledge among elderly Bedouin women in the Negev: Memory, practice, and intergenerational transmission. J. Cross Cult. Gerontol..

[27] Burnette C.E., Cannon C. (2014). It will always continue unless we can change something: Consequences of intimate partner violence for Indigenous women, children, and families. Eur. J. Psychotraumatol..

